# Supplementary material for: Ameliorative Effects of Bifidobacterium animalis subsp. lactis J-12 on Hyperglycemia in Pregnancy and Pregnancy Outcomes in a High-Fat-Diet/Streptozotocin-Induced Rat Model
Source: Nutrients. 2022 Dec 29;15(1):170. doi: 10.3390/nu15010170 (PMC9824282; doi:10.3390/nu15010170)
Supplement: Supplementary file 1 [file nutrients-15-00170-s001.zip › nutrients-2059839-supplementary.pdf]

Supplementary material

Table S1 Effect of J-12 on the pancreatic tissue damage in rats with hyperglycemia in pregnancy.

Histopathological changes	Group score				
	CK	M	I	J-12	J+I
Heterogeneous distribution of islet cells	-	+	++	+	++
Decreased number of islet cells	-	+	++	+	++
Degenerated and irregular islet cells	-	++	++	++	++
Interlobular ductal dysplasia	-	-	-	-	-
Vacuolar degeneration	-	-	-	-	-
Particle denaturation	-	-	-	-	-
steatosis	-	-	-	-	-
Glycogen deposition	-	-	-	-	-
Vascular basement membrane thickening	-	-	-	-	-
Inflammatory cell infiltration	-	-/+	-	-	-

(-) not present, (-/+) few< "10%", (+) moderate and (++) extensive.

Table S2 Effect of J-12 on the hepatic tissue damage in rats with hyperglycemia in pregnancy.

Histopathological changes	Group score				
	CK	M	I	J-12	J+I
Structural abnormalities of the hepatic lobule	-	-	-	-	-
Unclear cell boundaries	-	-	-	-	-
Abnormally accumulated fat	-	-	-	-	-
vesicular steatosis	-	-	-	-	-
Fat vacuoles	-	+	-	-	-
Particle denaturation	-	-/+	-/+	-/+	-/+
Glycogen deposition	-	-	-	-	-
Vascular basement membrane thickening	-	-/+	-	-	-
Inflammatory cell infiltration	-	-/+	-	-	-/+

(-) not present, (-/+) few< "10%", (+) moderate and (++) extensive.

Table S3 Effect of J-12 on cardiac tissue damage of fetuses.

Histopathological changes	Group score				
	CK	M	I	J-12	J+I
Cellular nuclear sequestration	-	-/+	-	-	-
Cytoplasmic hydrological alterations	-	++	+	+	++
Chromatin hydrolysis of the nucleus	-	-	+	-	-
Cytoplasmic vacuolization	-	++	-/+	-/+	+
No apparent mitosis	-/+	-	-/+	-/+	-
Irregular transverse band of cardiomyocytes	-	++	-/+	-/+	+

(-) not present, (-/+) few< "10%", (+) moderate and (++) extensive.

Table S4 Effect of J-12 on renal tissue damage of fetuses.

Histopathological changes	Group score				
	CK	M	I	J-12	J+I
Glomerular shrinkage	-	-/+	-	-	-
Erythrocyte space increase	-	+	-/+	-/+	-/+
Formation of tubular vacuoles	-	+	-/+	-/+	-/+
Degeneration of proximal tubules	-	+	-/+	-/	-/+
Degeneration of distal convoluted tubules	-	+	-/+	-/+	-/+

(-) not present, (-/+) few< "10%", (+) moderate and (++) extensive.

Table S5 Effect of J-12 on hepatic tissue damage of fetuses.

Histopathological changes	Group score				
	CK	M	I	J-12	J+I
Structural abnormalities of the hepatic lobule	-	++	+	+	+
Unclear cell boundaries	-	-	-/+	-	-
Abnormally accumulated fat	-	-	-	-	-
vesicular steatosis	-	-	-	-	-
Fat vacuoles	-	-	-	-	-
Particle denaturation	-	+	+	-/+	-
Glycogen deposition	-	-	-	-	-
Vascular basement membrane thickening	-	+	-	-	-

(-) not present, (-/+) few< "10%", (+) moderate and (++) extensive.

Table S6 Effect of J-12 on placental tissue damage in rats with hyperglycemia in pregnancy.

Histopathological changes	Group score				
	CK	M	I	J-12	J+I
Basal layer					
Cystic degeneration of glycogen cells	-	++	-	-/+	-/+
Necrosis of spongiotrophoblast cells	-	+	-/+	-	-
Congestion of maternal arterial canals	-	-	-	-	-
Inflammatory infiltration of leukocytes	-	-	-	-	-
Cytolysis of giant cells	-	-	-	-/+	-/+
Labyrinth layer					
Disruption of trophoblastic cells	-	++	-	-	+
Endothelial to mesenchymal transition (glycogen cells formation)	-	-	-	-	-
Constriction of fetal vessels	-	++	+	-	-
Vasogenic edema	-	-	-	-	+
Thickening of the interhemal	-	-	-	-	-

(-) not present, (-/+) few< "10%", (+) moderate and (++) extensive.
